# Prognostic impact and predictive model for early non-curative recurrence after liver resection for hepatocellular carcinoma: a retrospective cohort study

**DOI:** 10.1097/JS9.0000000000003534

**Published:** 2025-10-06

**Authors:** Jun Shibamoto, Michihisa Moriguchi, Akifumi Notsu, Keita Mori, Ryo Ashida, Katsuhisa Ohgi, Shimpei Otsuka, Yoshiyasu Kato, Hideyuki Dei, Katsuhiko Uesaka, Teiichi Sugiura

**Affiliations:** a Division of Hepato-Biliary-Pancreatic Surgery, Shizuoka Cancer Center, Shizuoka, Japan; b Division of Interventional Radiology, Shizuoka Cancer Center, Shizuoka, Japan; c Department of Molecular Gastroenterology and Hepatology, Graduate School of Medical Science, Kyoto Prefectural University of Medicine, Kyoto, Japan; d Clinical Research Center, Shizuoka Cancer Center, Shizuoka, Japan

**Keywords:** early recurrence, hepatocellular carcinoma, liver resection, nomogram, non-curative recurrence, prognostic model

## Abstract

**Background:**

The prognostic impact of early non-curative recurrence after liver resection for hepatocellular carcinoma (HCC) and its predictive factors remain unknown. We aimed to identify independent predictive factors for early non-curative recurrence, develop a predictive model, and evaluate its clinical utility.

**Materials and Methods:**

We retrospectively analyzed 624 patients who underwent curative HCC resection between 2002 and 2020. Patients were randomly assigned to training (*n* = 416) and validation (*n* = 208) sets. Early recurrence was defined as recurrence within 2 years after surgery. Non-curative recurrence was defined as recurrence corresponding to the intermediate or advanced stage of the Barcelona Clinic Liver Cancer staging system based on tumor factors. A nomogram was constructed based on independent predictive factors identified via multivariate analysis. Model performance was assessed by the area under the receiver operating characteristic curve (AUC), calibration plots, and decision curve analysis.

**Results:**

Early non-curative recurrence was an independent prognostic factor for overall survival (OS) after surgery and OS after recurrence (hazard ratio = 5.387 and 2.806, *P* <0.001). Median survival time after surgery was significantly shorter in the early non-curative recurrence group than in the other group (training set: 34.7 vs. 178.6 months, *P* <0.001; validation set: 27.7 vs. 156.1 months, *P* <0.001). Five factors were independently associated with early non-curative recurrence: alpha-fetoprotein, tumor size, number of tumors, portal invasion, and serosal invasion. The predictive model demonstrated high calibration performance in the training (AUC = 0.825) and validation (AUC = 0.792) sets. Decision curve analysis confirmed the clinical utility of the predictive model.

**Conclusion:**

We developed and validated a novel predictive model for early non-curative recurrence, which is significantly associated with poor prognosis after curative liver resection for HCC. Its application may enhance individualized postoperative surveillance and treatment strategies, potentially improving long-term outcomes.

**HIGHLIGHTS**

- Early non-curative recurrence is an independent prognostic factor for overall survival after liver resection for hepatocellular carcinoma.
- A predictive model incorporating five independent factors – alpha-fetoprotein level, tumor size, number of tumors, portal vein invasion, and serosal invasion – was developed and validated.
- The model demonstrated excellent discriminative performance and calibration in both training and validation sets.
- Decision curve analysis confirmed the clinical utility of the model for individualized patient risk assessment.
- The model supports risk-adapted postoperative surveillance and adjuvant therapy strategies to improve survival outcomes.

## Introduction

Liver cancer ranks as the sixth most common malignancy and the third leading cause of cancer-related death worldwide^[1]^. Hepatocellular carcinoma (HCC) accounts for 75–85% of primary liver cancers and has a high recurrence rate – up to 70% within 5 years – even after curative liver resection^[1–3]^. Despite extensive investigation into systemic and locoregional treatment modalities, including systemic chemotherapy, transarterial chemoembolization (TACE), selective internal radiation therapy, antiangiogenic agents, and immunotherapy, these interventions have yet to demonstrate a marked improvement in the overall survival (OS) of patients with HCC^[4–8]^.

Recently, the IMbrave050 trial evaluated patients at high risk of recurrence after curative liver resection or ablation for HCC^[9]^. Early recurrence is strongly associated with poor OS^[2,10]^, and the trial demonstrated a reduction in the incidence of early recurrence with adjuvant atezolizumab plus bevacizumab compared with active surveillance. However, this regimen did not achieve OS superiority. These findings suggest that among patients with recurrence in the active surveillance group, a subgroup with relatively favorable prognoses existed and may have been able to receive curative-intent treatment. This could partially explain why the recurrence-free survival benefit from adjuvant therapy did not translate into an OS benefit.

In interpreting these results, recurrence patterns and curability at recurrence must be considered. Unlike many cancers, HCC may still be amenable to curative treatment at recurrence, depending on disease pattern and patient condition at the time of recurrence. Previous studies have shown that patients who undergo curative treatment for recurrence achieve substantially better long-term outcomes^[11,12]^. Conversely, patients with non-curative recurrence face limited therapeutic options and poorer survival. We therefore hypothesized that among patients with early recurrence after curative liver resection for HCC, patients with non-curative recurrence exhibit markedly inferior OS compared with other recurrence patterns. Furthermore, identifying predictive factors and developing a predictive model for early non-curative recurrence could improve survival outcomes for patients with HCC.

Understanding the biological characteristics of primary HCC is essential for predictive model development. Clearly distinguishing between intrahepatic metastasis and *de novo* tumor formation in recurrent HCC is challenging. Clinically, this distinction is typically based on recurrence timing: early recurrence is generally attributed to intrahepatic metastases from the primary tumor, whereas late recurrence is associated with *de novo* tumorigenesis related to underlying liver disease^[13,14]^. The most widely used cutoff between early and late recurrence is 2 years^[13–15]^. This distinction aligns with the biological characteristics of primary HCC and enhances the predictive model’s accuracy.

Although early non-curative recurrence is a critical determinant of long-term prognosis after liver resection for HCC, few studies have examined this intersection in detail. Risk stratification for early non-curative recurrence could improve the precision of postoperative surveillance. Moreover, identifying patients at high risk could guide intensified follow-up strategies and refine patient selection for adjuvant therapies trials.

Accordingly, we aimed to investigate the prognostic impact of early non-curative recurrence after curative liver resection for HCC, to develop and validate a predictive model for identifying high-risk patients, and to examine its potential for clinical application.

## Methods

### Patients

We retrospectively reviewed a prospectively maintained database of patients undergoing liver resection for HCC. Among the 810 patients who underwent liver resection between September 2002 and December 2020, 186 were excluded: 110 received prior treatment, 46 had other primary cancers, 26 were pathologically diagnosed with residual cancer, and 4 experienced in-hospital mortality within 30 days postoperatively. The final cohort included 624 patients. The analysis cohort was randomly divided into training and validation sets using the RAND function in Microsoft Excel (Microsoft Corporation, Redmond, WA, USA).

This study complied with the Declaration of Helsinki and was approved by the Institutional Review Board (institutional approval no. J2024-149-2024-1-3). Participants were notified about the study and given the opportunity to opt out of participation. For minors, parents or legal guardians were informed about the study and could withdraw their child from participation at any time. Minors were also informed when appropriate and could decline participation. This type of research is generally authorized to be conducted with this approach in Japan. This study is reported following the STROCSS guidelines^[16]^.

### Preoperative assessment

All patients underwent comprehensive preoperative assessment, including physical examination, laboratory testing, electrocardiography, pulmonary function testing, liver function testing, and imaging to evaluate exercise tolerance, nutritional status, organ function, and tumor characteristics.

Liver function was evaluated using the Child–Pugh classification^[17]^; patients with a Child–Pugh classification of C were considered ineligible for resection. Liver functional reserve was assessed using the plasma clearance rate of indocyanine green and future liver remnant volume. Preoperative albumin–bilirubin (ALBI) score was calculated as: ALBI score = log_10_(bilirubin [μmol/L]) × 0.66 + albumin [g/L] × −0.085. Grades were assigned as follows: grade 1 for scores ≤−2.60, grade 2 for >−2.60 to ≤− 1.39, and grade 3 for >−1.39^[18]^.

### Surgery and postoperative complications

The surgical approach was determined in a multidisciplinary conference, considering tumor location, tumor size, extension to surrounding structures, and residual liver function. Technical details have been described previously^[19]^.

Liver resection was defined as minor (≤2 segments) or major (≥3 segments) per the Brisbane 2000 glossary^[20]^. Postoperative complications were recorded and graded using the Clavien–Dindo classification^[21]^. Postoperative bile leakage was classified according to the International Study Group of Liver Surgery definition^[22]^.

### Follow-up and treatment strategy for recurrence

Postoperative follow-up was performed every 3–6 months, including physical examination, blood tests, and imaging. Recurrence was diagnosed radiologically.

Treatment decisions for recurrent HCC were based on recurrence pattern, tumor size and number, anatomical location, liver function, time to recurrence, and performance status. In general, the same treatment algorithm used for initial HCC diagnosis was applied^[23]^.

For intrahepatic recurrence, repeat liver resection or percutaneous local ablation therapy (radiofrequency ablation, percutaneous ethanol injection, and percutaneous microwave coagulation therapy) was considered for small tumors (≤3 cm) and a limited number of tumors (≤3). For multiple intrahepatic lesions (≥4), TACE was preferred, with hepatic arterial infusion chemotherapy considered for select cases.

For extrahepatic recurrence, systemic therapy became the standard after sorafenib was approved in Japan in 2009^[24]^. Since then, additional first-line options – lenvatinib and atezolizumab plus bevacizumab – have been introduced^[25,26]^. Choice of agents considered tumor status, liver function, performance status, age, comorbidities, and temporal context.

If intrahepatic lesions were controlled or absent and extrahepatic lesions were solitary and resectable, metastasectomy was performed. Bone or brain metastases were treated with external-beam radiotherapy in selected patients.

### Pathological diagnosis

Specimens were fixed in 10% formalin for at least 48 h, serially sectioned, embedded in paraffin, and stained with hematoxylin–eosin. All cases were pathologically confirmed as HCC.

Tumor characteristics evaluated included size, number, histological grade, portal vein invasion, hepatic vein invasion, bile duct invasion, growth pattern, fibrous capsule, and serosal invasion. Portal vein, hepatic vein, and bile duct invasion were considered present when microscopic invasion was confirmed through histopathological examination of the resected specimens. Serosal invasion was diagnosed on pathological examination of hematoxylin–eosin-stained paraffin sections from the resected specimens, with independent assessments performed by two experienced pathologists. Pathological stage was assigned according to the eighth edition of the Union for International Cancer Control classification^[27]^.

### Study definitions and cutoff values

Early recurrence was defined as initial recurrence within 2 years; late recurrence as recurrence after 2 years^[13–15]^. Early recurrence was subclassified into curative or non-curative recurrence. Patients with early curative recurrence who developed non-curative recurrence within 2 years after initial liver resection were classified as early non-curative recurrence.

Curative and non-curative recurrence were defined using tumor factors from the Barcelona Clinic Liver Cancer (BCLC) staging system^[23]^. Curative recurrence refers to very early (0) or early stage (A); non-curative recurrence to intermediate (B) or advanced stage (C). Specifically, curative recurrence met the following criteria: intrahepatic lesions only, with (1) a single lesion or (2) two to three lesions, each ≤3 cm in diameter. Non-curative recurrence met any of the following criteria: (1) intrahepatic lesions with (a) four or more lesions or (b) two to three lesions with at least one lesion >3 cm in diameter, (2) portal vein invasion; or (3) extrahepatic spread.

Based on previous reports associated with early recurrence of HCC after liver resection, cutoff values for alpha-fetoprotein (AFP) level and tumor size were set at 20 ng/mL and 50 mm, respectively^[28–30]^. These values were used in univariate and multivariate analyses to identify prognostic factors for OS and predictors of early non-curative recurrence.

### Statistical analysis

Statistical analyses were performed using R version 4.4.2 (R Foundation for Statistical Computing, Vienna, Austria) and JMP version 18 (SAS Institute, Cary, NC, USA). Continuous variables are presented as medians with interquartile ranges and compared using the Mann–Whitney *U-*test. Categorical variables were compared using Fisher’s exact test or Pearson’s χ^2^ test. OS was calculated using the Kaplan–Meier method from either the date of surgery (OS after surgery) or recurrence (OS after recurrence) to death from any cause, with the last follow-up as the censoring point. Survival differences were measured using the log-rank test. Cox proportional hazards regression was used for univariate and multivariate analyses of prognostic factors for OS. Logistic regression was used to identify predictors of early non-curative recurrence. Variables significant in univariate analysis were included in the multivariate model. All tests were two-sided, with *P* <0.05 considered significant.

### Nomogram development and model evaluation

Independent predictors from the multivariate analysis were used to develop a nomogram with the rms package in R. All variables were reviewed for completeness and clinical relevance; no imputation was required as there were no missing data. Continuous variables were entered without normalization or transformation, as the logistic regression model does not assume normally distributed predictors. A logistic regression model was constructed using the lrm function in the rms package, with early non-curative recurrence as the outcome. Predictors included serum AFP level, tumor size, number of tumors, portal vein invasion, and serosal invasion, based on their clinical relevance and univariate associations. The nomogram function was used to generate the model, assigning weighted point scores proportional to regression coefficients. Total points were then mapped to the corresponding predicted probabilities of early non-curative recurrence using the inverse logit (plogis) function. The datadist function was used to define variable distributions and ensure proper scaling in the nomogram.

Model performance was assessed using the area under the receiver operating characteristic (ROC) curve (AUC) for discrimination, calibration plots, and the Hosmer–Lemeshow test for goodness-of-fit^[31]^. Internal validation was conducted. Decision curve analysis was used to assess net clinical benefit at different probability thresholds, compared with each variable, combined variables, and default strategies of treating all patients or none.

## Results

### Patient allocation and grouping

Supplementary Digital Content Fig. 1, available at: http://links.lww.com/JS9/F190 presents a flowchart of patient selection for this study. The analysis cohort was randomly divided in a 2:1 ratio into training (*n* = 416) and validation (*n* = 208) sets. Within each set, patients were classified into the early non-curative recurrence group and the other group. The other group included patients with no recurrence, late recurrence, or early curative recurrence. In the training and validation sets, the early non-curative recurrence group comprised 112 and 59 patients, respectively, while the other group comprised 304 and 149 patients, respectively.

### Patient characteristics

The clinicopathological characteristics of the cohort are presented in Table 1. In the overall cohort, the median age was 70 years, the median serum AFP level was 13.9 ng/mL, and 58.3% of patients had a history of viral infection. Baseline characteristics were generally comparable between the training and validation sets.

**Table 1:** Clinicopathological characteristics of patients

	Analysis cohort		Training set			Validation set	
Variable	*n* = 624		*n* = 416			*n* = 208	
*Preoperative factors*							
Age, yr	70	(63–75)	70	(63–75)		70	(63–76)
Sex, Male, n (%)	496	(79.5)	333	(80.1)		163	(78.4)
ECOG PS, 0/1	611/13		408/8			203/5	
Body mass index, kg/m^2^	22.8	(20.8–25.0)	22.7	(20.8–24.8)		22.9	(20.7–25.1)
Etiology, Non-virus/HBV/HCV/HBV + HCV	260/119/234/11		165/81/163/7			95/38/71/4	
Child-Pugh classification, A/B	607/17		406/10			201/7	
ALBI score	−2.905	(−3.112– − 2.676)	−2.926	(−3.125– − 2.688)		−2.904	(−3.075– − 2.606)
AFP, ng/mL	13.9	(4.7–216.3)	13.0	(4.8–216.3)		16.0	(4.7–212.5)
BCLC stage, 0/A/B/C	77/441/67/39		52/290/48/26			25/151/19/13	
*Surgical factors and outcomes*							
Year of surgery, 2002–2011/2012–2022	287/337		191/225			96/112	
Major liver resection, ≥3 segments, n (%)	194	(31.1)	124	(29.8)		70	(33.7)
Blood transfusion, n (%)	92	(14.7)	66	(15.9)		26	(12.5)
Postoperative complication, C-D ≥IIIa, n (%)	87	(13.9)	60	(14.4)		27	(13.0)
Bile leakage, ISGLS Grade ≥B, n (%)	60	(9.6)	43	(10.3)		17	(8.2)
*Pathological factors*							
Longest diameter of the largest tumor, mm	37	(25–70)	36	(25–65)		39	(25–75)
Number of tumors	1	(1–1)	1	(1–1)		1	(1–1)
Histological grade^a^, G1/2, n (%)	604	(96.8)	403	(96.9)		201	(96.6)
Portal vein invasion, n (%)	151	(24.2)	102	(24.5)		49	(23.6)
Hepatic vein invasion, n (%)	55	(8.8)	34	(8.2)		21	(10.1)
Bile duct invasion, n (%)	21	(3.4)	13	(3.1)		8	(3.9)
Growth pattern, Expansive/Invasive	601/23		401/15			200/8	
Fibrous capsule, n (%)	520	(83.3)	343	(82.5)		177	(85.1)
Serosal invasion, n (%)	39	(6.3)	26	(6.3)		13	(6.3)

Continuous variables are presented as medians, and interquartile ranges are shown in parentheses.

*ECOG PS,* Eastern Cooperative Oncology Group Performance Status; *HBV,* Hepatitis B virus; *HCV,* Hepatitis C virus; *ALBI,* Albumin-bilirubin; *AFP,* Alpha-fetoprotein;

*BCLC,* Barcelona Clinic Liver Cancer; *C–D,* Clavien–Dindo classification; *ISGLS,* International Study Group of Liver Surgery

^a^ According to the eighth edition of the Union for International Cancer Control staging

### Survival outcomes according to recurrence pattern

The median follow-up duration for censored cases was 75.5 months (interquartile range: 50.8–116.3). Supplementary Digital Content Fig. 2, available at: http://links.lww.com/JS9/F191 shows OS by recurrence pattern in the analysis cohort. OS after surgery was significantly worse in the early non-curative recurrence group compared with the other three groups (Supplementary Digital Content Fig. 2A, available at: http://links.lww.com/JS9/F191). Among patients with recurrence (excluding those without recurrence), OS after recurrence was also significantly worse in the early non-curative recurrence group compared with the early curative recurrence and late recurrence groups (Supplementary Digital Content Fig. 2B, available at: http://links.lww.com/JS9/F191). There was no significant difference in OS after recurrence between the early curative recurrence and late recurrence groups (median survival time [MST]: 94.8 vs. 75.2 months; *P* = 0.154).

**Figure 1.**
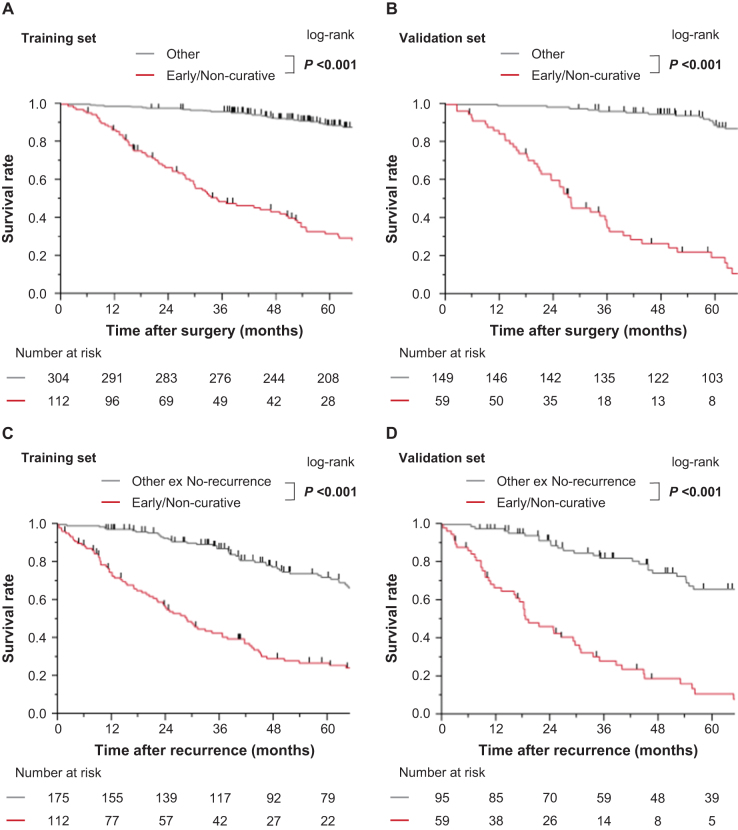
Kaplan–Meier curves show survival outcomes based on the presence or absence of early non-curative recurrence. Kaplan–Meier curves of OS after surgery in (A) the training and (B) validation sets. Kaplan–Meier curves of OS after recurrence in (C) the training and (D) validation sets. The differences were calculated using the log-rank test. OS, overall survival; Other ex No-recurrence, Other group excluding no-recurrence group.

In the training set, OS after surgery was significantly shorter in the early non-curative recurrence group than in the other group (MST: 34.7 vs. 178.6 months, *P* <0.001; Fig. 1A), and the same pattern was observed in the validation set (MST: 27.7 vs. 156.1 months, *P* <0.001; Fig. 1B). For OS after recurrence (excluding patients without recurrence), the early non-curative recurrence group had significantly shorter survival than the other group in both the training set (MST: 28.7 vs. 82.9 months; *P* <0.001; Fig. 1C) and validation set (MST: 19.1 vs. 93.2 months; *P* <0.001; Fig. 1D). These findings underscore the prognostic importance of distinguishing early non-curative recurrence from other recurrence patterns.

### Prognostic factors

Table 2 summarizes the univariate and multivariate analyses for OS after surgery and OS after recurrence in the training set. Independent prognostic factors for OS after surgery included early non-curative recurrence (hazard ratio [HR] = 5.387; *P* <0.001), age (HR = 2.316; *P* <0.001), portal vein invasion (HR = 1.629; *P* = 0.008), blood transfusion (HR = 1.521; *P* = 0.039), and multiple tumors (HR = 1.514; *P* = 0.019). For OS after recurrence, early non-curative recurrence (HR = 2.806; *P* <0.001) and age (HR = 2.005; *P* <0.001) were independent prognostic factors. Early non-curative recurrence was consistently associated with worse survival, conferring more than a fivefold increase in risk of death after surgery and nearly threefold increased risk after recurrence, representing clinically significant survival disadvantages.

**Table 2:** Univariate and multivariate analyses associated with overall survival in training set

			OS after surgery						OS after recurrence				
			Univariate		Multivariate				Univariate		Multivariate		
Variable			*P*		HR	(95% CI)	*P*		*P*		HR	(95% CI)	*P*
Age, yr	≥70 vs. <70		**<0.001**		2.316	1.660–3.244	**<0.001**		**0.003**		2.005	1.421–2.838	**<0.001**
Sex	Male vs. Female		0.216						0.735				
ECOG PS	1 vs. 0		0.420						0.704				
Body mass index, kg/m^2^	<25 vs. ≥25		0.324						0.932				
Etiology	Non-virus vs. Virus		0.581						0.311				
ALBI grade	2/3 vs. 1		**0.018**		1.307	0.873–1.913	0.189		0.216				
AFP, ng/mL	>20 vs. ≤20		**<0.001**		1.202	0.861–1.681	0.280		**0.004**		1.266	0.890–1.803	0.189
Blood transfusion	Present vs. Absent		**<0.001**		1.521	1.023–2.212	**0.039**		**0.007**		1.430	0.936–2.131	0.097
Postoperative complication	C-D ≥IIIa vs. C-D ≤II		0.372						0.392				
Tumor size, mm	>50 vs. ≤50		**<0.001**		1.193	0.841–1.681	0.320		**<0.001**		1.200	0.841–1.704	0.313
Number of tumors	Multiple vs. Single		**<0.001**		1.514	1.071–2.125	**0.019**		**0.019**		1.222	0.871–1.706	0.244
Histological grade^a^	G3/4 vs. G1/2		0.958						0.633				
Portal vein invasion	Present vs. Absent		**<0.001**		1.629	1.141–2.301	**0.008**		**0.002**		1.382	0.959–1.968	0.082
Hepatic vein invasion	Present vs. Absent		**0.010**		1.182	0.718–2.048	0.524		0.082				
Bile duct invasion	Present vs. Absent		0.287						0.972				
Growth pattern	Invasive vs. Expansive		0.117						0.084				
Fibrous capsule	Present vs. Absent		0.193						0.182				
Serosal invasion	Present vs. Absent		**0.001**		1.679	0.953–2.785	0.071		**0.038**		1.375	0.757–2.326	0.280
Recurrence	Early non-curative recurrence vs. Other		**<0.001**		5.387	3.761–7.716	**<0.001**		**<0.001**		2.806	1.962–4.026	**<0.001**

Significant values are given in boldface.

Univariate and multivariate analyses for OS after recurrence were performed in patients with recurrence.

*OS,* Overall survival; *HR,* Hazard ratio; *CI,* Confidence interval; *ECOG PS,* Eastern Cooperative Oncology Group Performance Status; *ALBI,* Albumin-bilirubin; *AFP,* Alpha-fetoprotein; *C–D,* Clavien–Dindo classification

^a^ According to the eighth edition of the Union for International Cancer Control staging

### Predictive factors for early non-curative recurrence

Multivariate analysis identified five independent predictive factors for early non-curative recurrence (Table 3): multiple tumors (odds ratio [OR] = 3.914; *P* <0.001), large tumor size (OR = 3.455; *P* <0.001), high AFP level (OR = 2.815; *P* <0.001), serosal invasion (OR = 2.629; *P* = 0.046), and portal vein invasion (OR = 1.992; *P* = 0.017). These factors were associated with approximately 4-, 3.5-, 3-, 2.5-, and 2-fold increased risks, respectively.

**Table 3:** Univariate and multivariate analyses associated with early non-curative recurrence in training set

		Other		Early non-curative recurrence		Univariate		Multivariate		
Variable		*n* = 304		*n* = 112		*P*		OR	(95% CI)	*P*
Age, yr	≥70 vs. <70	155/149		57/55		0.986				
Sex	Male vs. Female	241/63		92/20		0.516				
ECOG PS	1 vs. 0	6/298		2/110		1.000				
Body mass index, kg/m^2^	<25 vs. ≥25	230/74		85/27		0.961				
Etiology	Non-virus vs. Virus	120/184		45/67		0.896				
Child-Pugh classification	A vs. B	296/8		110/2		1.000				
ALBI grade	2/3 vs. 1	50/254		29/83		**0.029**		1.327	0.687–2.512	0.395
AFP, ng/mL	>20 vs. ≤20	109/195		77/35		**<0.001**		2.815	1.671–4.801	**<0.001**
Blood transfusion	Present vs. Absent	36/268		30/82		**<0.001**		1.387	0.716–2.640	0.328
Postoperative complication	C-D ≥IIIa vs. C-D ≤II	46/258		14/98		0.498				
Tumor size, mm	>50 vs. ≤50	69/235		67/45		**<0.001**		3.455	2.029–5.927	**<0.001**
Number of tumors	Multiple vs. Single	66/238		61/51		**<0.001**		3.914	2.294–6.752	**<0.001**
Histological grade^a^	G3/4 vs. G1/2	7/297		6/106		0.112				
Portal vein invasion	Present vs. Absent	53/251		49/63		**<0.001**		1.992	1.132–3.484	**0.017**
Hepatic vein invasion	Present vs. Absent	16/288		18/94		**<0.001**		1.019	0.419–2.515	0.967
Bile duct invasion	Present vs. Absent	7/297		6/106		0.112				
Growth pattern	Invasive vs. Expansive	9/295		6/106		0.245				
Fibrous capsule	Present vs. Absent	247/57		96/16		0.288				
Serosal invasion	Present vs. Absent	10/294		16/96		**<0.001**		2.629	1.017–6.966	**0.046**

Significant values are given in boldface.

*OR,* Odds ratio; *CI,* Confidence interval; *ECOG PS,* Eastern Cooperative Oncology Group Performance Status; *ALBI,* Albumin-bilirubin; *AFP,* Alpha-fetoprotein; *C–D,* Clavien–Dindo classification.

^a^ According to the eighth edition of the Union for International Cancer Control staging

### Nomogram development and predictive model evaluation

**Figure 2.**
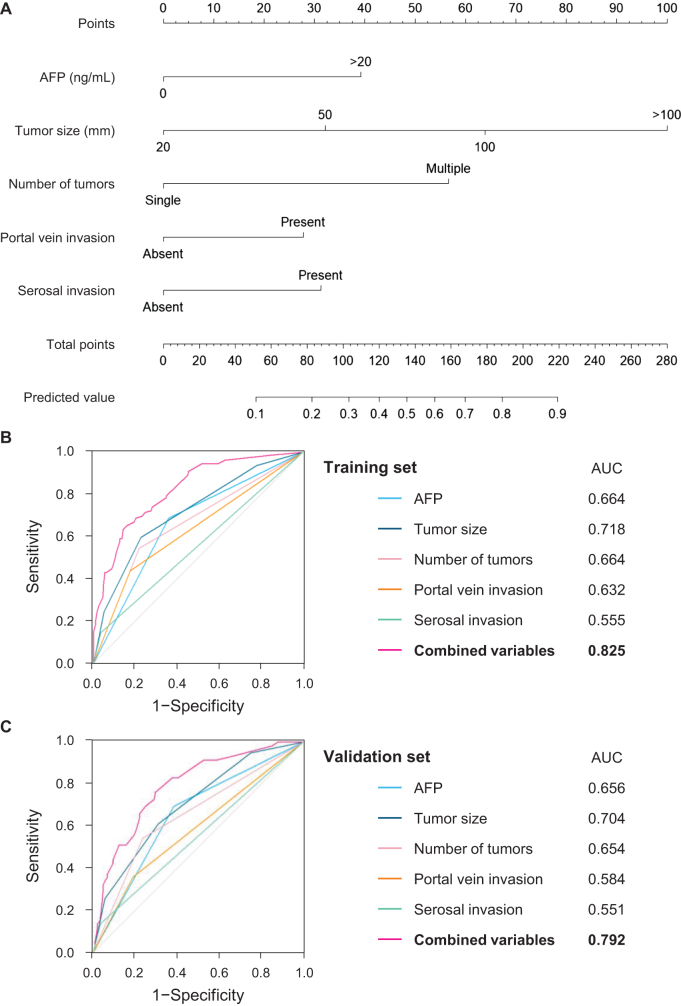
Nomogram, ROC curves, and AUCs. (A) Nomogram predicting the risk of early non-curative recurrence using independent predictive factors identified in the multivariate analysis of the training set. (B, C) The ROC curves and AUCs in (B) the training and (C) validation sets. ROC, receiver-operating characteristic; AUC, area under the receiver-operating characteristic curve; AFP, alpha-fetoprotein.

A nomogram for predicting early non-curative recurrence after curative liver resection for HCC was constructed based on independent predictive factors identified in the multivariate analysis of the training set (Fig. 2A). Model discrimination was evaluated using the AUC, with ROC curves presented for individual and combined variables in the training (Fig. 2B) and validation (Fig. 2C) sets. The AUCs for combined variables were 0.825 (95% confidence interval [CI]: 0.781–0.869) in the training set and 0.792 (95% CI: 0.726–0.859) in the validation set.

**Figure 3.**
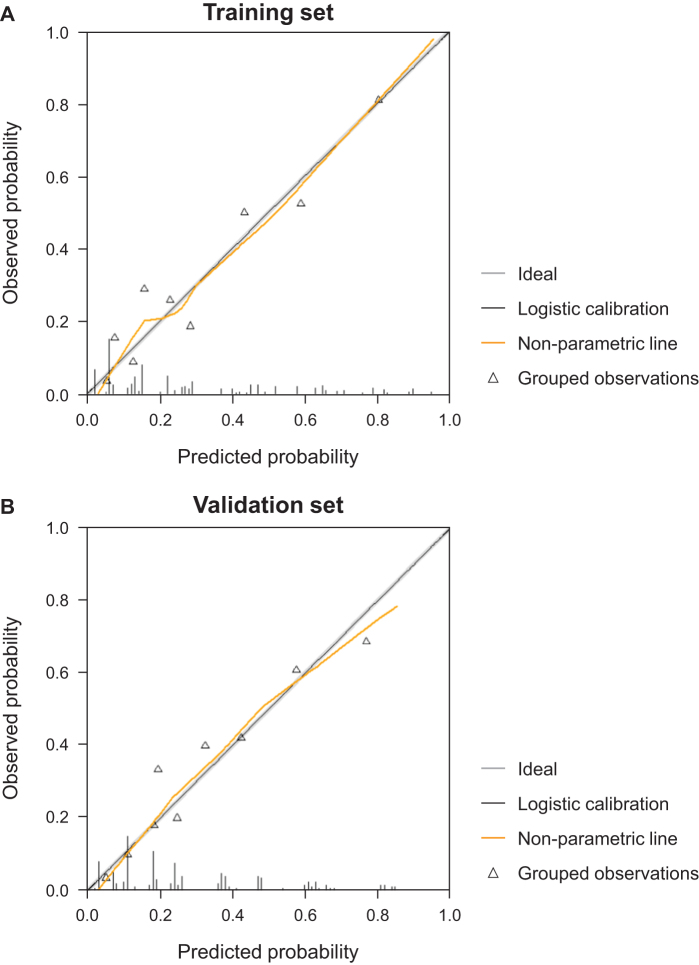
Calibration plots of the predictive model for early non-curative recurrence in (A) the training and (B) validation sets.

Calibration plots (Fig. 3A, B) demonstrated close agreement between predicted probabilities and observed early non-curative recurrence rates, with points aligning closely to the ideal 45° reference line in both sets. Non-significant *P*-values for the Hosmer–Lemeshow test (training set: *P* = 0.858; validation set: *P* = 0.964, respectively) confirm good calibration.

### Decision Curve Analysis

**Figure 4.**
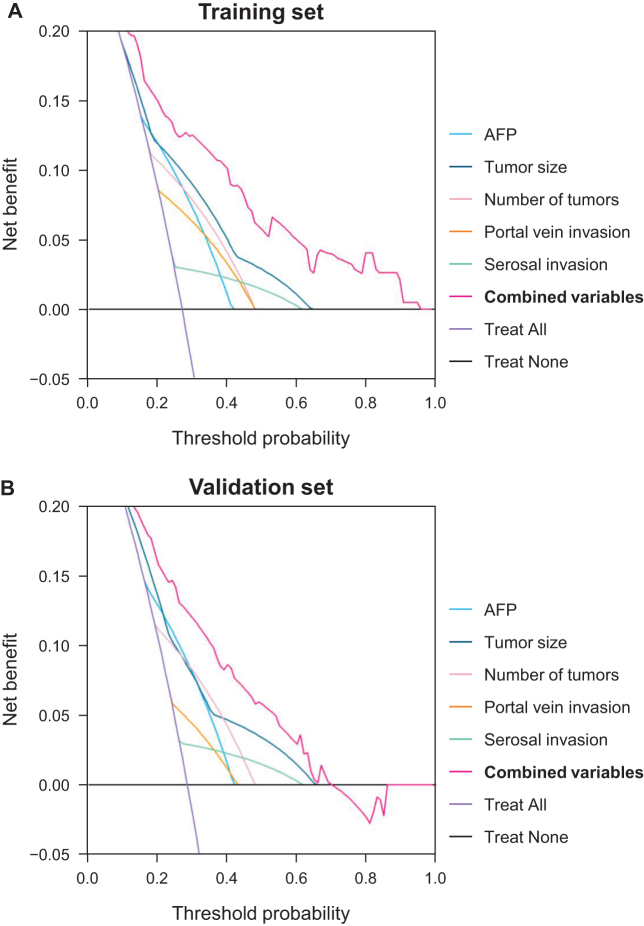
Decision curve analysis comparing each variable, combined variables, and default strategies (treating all patients or none) in (A) the training and (B) validation sets. AFP, alpha-fetoprotein.

Decision curve analysis was used to evaluate the clinical utility of the predictive model (Fig. 4). Across a range of threshold probabilities for recurrence risk, the model provided greater net benefit than any individual predictive variable, as well as strategies of treating all patients or none, in both the training (Fig. 4A) and validation (Fig. 4B) sets. This finding supports the model’s potential to guide postoperative decision-making and improve patient outcomes.

## Discussion

This study demonstrates that early non-curative recurrence is a distinct prognostic factor that markedly influences survival after curative liver resection for HCC. We developed and validated a predictive model and nomogram for early non-curative recurrence, incorporating five independent predictive factors: serum AFP level, tumor size, number of tumors, portal vein invasion, and serosal invasion. The model demonstrated strong calibration and discrimination in both the training and validation sets, and decision curve analysis confirmed its clinical utility. These findings provide a foundation for more precise postoperative management strategies aimed at improving long-term survival in patients with HCC after liver resection.

Our survival analyses reveal that the prognosis of patients with HCC is determined by the timing of recurrence and the feasibility of treatment at the time of recurrence. The similar survival outcomes between the early curative recurrence and late recurrence groups demonstrate a unique feature of HCC: even after recurrence, curative-intent treatment can yield long-term outcomes. This highlights the need to assess both recurrence timing and curative potential when evaluating prognosis.

Previous studies have identified early recurrence – commonly defined as recurrence within 2 years – as a poor prognostic indicator after liver resection for HCC^[2,10]^. The unfavorable outcomes in early recurrence are often attributable to the high incidence of multiple intrahepatic or extrahepatic lesions, which frequently preclude curative-intent treatment^[32]^. Our findings support the hypothesis that early non-curative recurrence is a critical determinant of survival, likely reflecting the aggressive tumor biology. Furthermore, considerable overlap may exist between oncological factors associated with early recurrence and non-curative recurrence.

The predictive factors for early recurrence identified in prior research – large tumor size, multiple tumors, serosal invasion, high histological grade, and high AFP level – are generally consistent with this study, linking them to aggressive tumor phenotypes^[32–35]^. While several studies have explored recurrence beyond the Milan criteria^[36,37]^, only a few have specifically addressed non-curative recurrence. The predictive factors identified in our study fill this gap, as has been shown in previous basic and clinical studies to be strongly associated with the biological aggressiveness of the primary tumor^[38–42]^, highlighting the relevance of these factors in predicting early non-curative recurrence.

The excellent discriminative performance and calibration of our predictive model in both the training and validation sets support its reliability. Decision curve analysis further demonstrated the potential clinical benefit of risk-based strategies^[43]^. For patients with high risk, intensified postoperative surveillance could enable earlier detection of recurrence while curative options remain available. Additionally, such patients may be suitable candidates for adjuvant therapy aimed at preventing non-curative recurrence and improving OS. Our model may thereby serve as a valuable tool for risk-adapted patient selection in clinical trials and for optimizing therapeutic decision-making.

Despite these promising findings, several limitations should be noted. First, this was a single-center retrospective study with a limited number of early non-curative recurrence cases, and external validation was not performed. Second, this study included patients with BCLC stage B and C HCC, which were classified as non-curative. We included BCLC stage B and C patients due to evolving historical practices and discrepancies between clinical and pathological staging, which justified their classification as having undergone curative-intent resection. Third, the long study period included evolving treatment approaches, which may have introduced heterogeneity in patient management. Fourth, treatment decisions for recurrence were made in a multidisciplinary conference, and some patients classified as non-curative by conventional criteria underwent curative-intent treatment following individualized assessment. This may have influenced survival outcomes. Finally, detailed analyses of postrecurrence treatment courses were not conducted. Future research should validate our findings in larger, multicenter cohorts using standardized treatment protocols.

## Conclusion

Early non-curative recurrence after curative liver resection for HCC is an independent prognostic factor for both OS after surgery and OS after recurrence. We developed and validated a predictive model using the following five independent predictive risk factors – serum AFP level, tumor size, number of tumors, portal vein invasion, and serosal invasion – that demonstrated excellent predictive performance. It enables personalized postoperative management by identifying high-risk patients who may benefit from intensified surveillance to detect recurrence earlier, when curative interventions remain feasible. Furthermore, this model provides a robust framework for risk-adapted patient selection in adjuvant therapy trials, potentially improving treatment allocation and patient outcomes. Integrating this predictive model into clinical practice may enable precision medicine approaches that optimize survival for patients with HCC after liver resection.

## Supplementary Material

**Figure:**
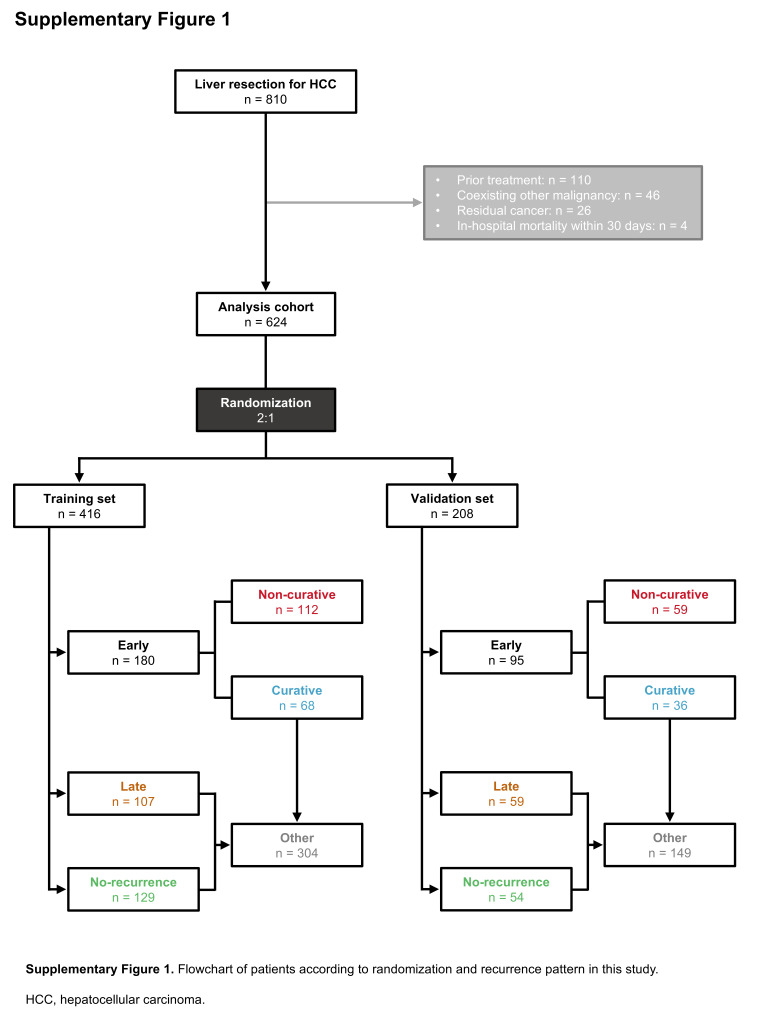


**Figure:**
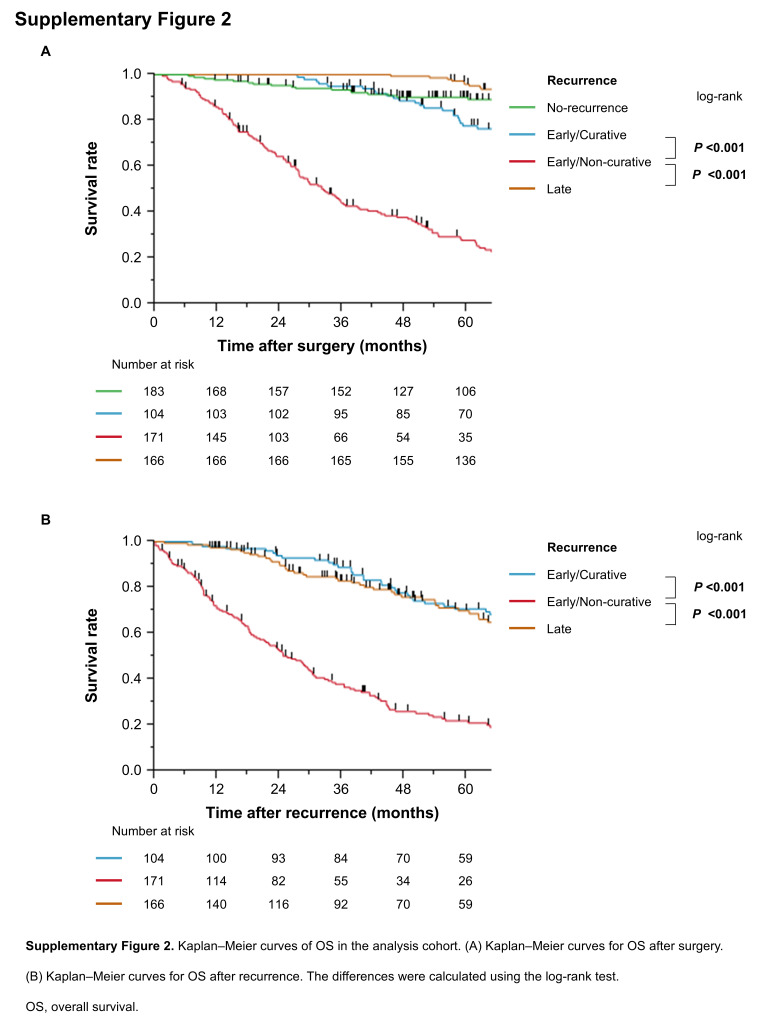


## Data Availability

The data in this study are available from the corresponding author on reasonable request.

## Research registration unique identifying number (UIN)

Name of the registry: Research Registry; Unique Identifying number or registration ID: researchregistry11258; Hyperlink to your specific registration (must be publicly accessible and will be checked):

https://www.researchregistry.com/browse-theregistry/# home/registrationdetails/68404b4562843602fe821bbd/
